# Relationship between Urinary Alzheimer-Associated Neuronal Thread Protein and Apolipoprotein Epsilon 4 Allele in the Cognitively Normal Population

**DOI:** 10.1155/2020/9742138

**Published:** 2020-06-06

**Authors:** Yuxia Li, Meimei Kang, Can Sheng, Guanqun Chen, Taoran Li, Jun Wang, Yanning Cai, Rong Wang, Ying Han

**Affiliations:** ^1^Department of Neurology, Xuanwu Hospital of Capital Medical University, Beijing, China; ^2^Central Laboratory, Xuanwu Hospital, Capital Medical University, Beijing, China; ^3^Department of Neurobiology, Xuanwu Hospital, Capital Medical University, Beijing, China; ^4^Beijing Geriatric Medical Research Center, Beijing, China; ^5^Center of Alzheimer's Disease, Beijing Institute for Brain Disorders, Beijing, China; ^6^National Clinical Research Center for Geriatric Disorders, Beijing, China

## Abstract

We investigated the relationship between urinary Alzheimer-associated neuronal thread protein (AD7c-NTP) levels and apolipoprotein epsilon 4 (*ApoE ɛ4*) alleles, as well as other factors that cause cognitive decline, in the cognitively normal population. We recruited 329 cognitively normal right-handed Han Chinese subjects who completed *ApoE* gene testing and urinary AD7c-NTP testing. There was no significant difference in urinary AD7c-NTP levels between the normal control and subjective cognitive decline groups. Urinary AD7c-NTP levels were significantly higher in subjects with *ApoE ɛ3/4* and *4/4* [0.6074 (0.6541) ng/mL] than in subjects without *ApoE ɛ4* [0.4368 (0.3392) ng/mL and 0.5287 (0.3656) ng/mL], and urinary AD7c-NTP levels positively correlated with *ApoE* genotype grade (*r* = 0.165, *p* = 0.003). There were significant differences in urinary AD7c-NTP levels between subjects with and without a history of coronary heart disease or diabetes. Urinary AD7c-NTP levels were not related to years of education, nature of work, family history of dementia, a history of hypertension, stroke, anemia, or thyroid dysfunction. Urinary AD7c-NTP levels were positively correlated with *ApoE* grade in the cognitively normal population. The relationship between risk factors of cognitive decline and urinary AD7c-NTP levels provides a new way for us to understand AD and urinary AD7c-NTP.

## 1. Introduction

Alzheimer's disease (AD) is the most common form of dementia, accounting for 60%–80% of all dementia, and imposes a substantial socioeconomic burden on society and families [1–4]. Furthermore, AD is an irreversible, disabling degenerative disease of the central nervous system. In the absence of curative treatments once the disease has progressed to AD dementia, primary prevention and early diagnosis in the preclinical stage of AD have been the main research focuses in recent years [5]. It is therefore crucial to explore early biomarkers of AD to enable the early diagnosis of this disease, with the ultimate aim of treating and preventing dementia in preclinical AD.

Urinary AD7c-NTP, a peripheral biomarker for AD, is increased in mild cognitive impairment (MCI) and AD [6–9]. Previous studies have reported that AD7c-NTP immunoreactivity colocalizes with neurofibrillary tangles and dystrophic neurites, and increased AD7c-NTP levels are associated with tau-immunoreactive neurofibrillary tangles [10]. Furthermore, overexpression of AD7c-NTP is associated with neurite sprouting and cell death, which is reflected in AD neurodegeneration [11]. Recent research suggests that urinary AD7c-NTP is increased in hypertensive patients with cognitive impairment [12]. In addition, a previous study reported that urinary AD7c-NTP is elevated in late-life depression with cognitive impairment [13]. Furthermore, existing research recognizes that urinary AD7c-NTP has a critical role in cognitive decline, but not in depression or other diseases [12–15]. Urinary AD7c-NTP testing is noninvasive, radiation-free, repeatable, and easy to carry out. Therefore, urinary AD7c-NTP may be a promising peripheral biomarker for detecting cognitive decline and disease progression [16]. Although previous studies have reported that cognitive decline is associated with elevated urinary AD7c-NTP, it is not clear which factors, aside from decreased cognitive function, are associated with elevated urinary AD7c-NTP [13, 15, 17, 18]. It has not been reported whether other medical history factors that may lead to a decline in cognitive function, such as carbon monoxide poisoning (COP), general anesthesia, or thyroid dysfunction, are associated with urinary AD7c-NTP levels in the normal cognitive population.

Subjective cognitive decline (SCD) refers to self-experienced persistent cognitive decline compared with a previously normal status, while standardized cognitive tests give objectively normal results [19, 20]. SCD is considered to be a preclinical phase of AD in patients who are objectively cognitively normal [21, 22]. Approximately 14.1% of SCD patients convert to AD within 4 years [21]. SCD-plus, proposed by the Subjective Cognitive Decline Initiative, is considered closer to the early stage of AD [20, 23]. The conversion rate for SCD-plus to MCI is 18.9% [22]. Therefore, both early diagnosis of AD and monitoring of progression in the SCD stage are essential for the early prevention and treatment of AD.

The apolipoprotein E epsilon 4 (*ApoE ɛ4*) allele is considered to be a risk factor for AD, and *ApoE ɛ4* may influence the rate of cognitive decline in early AD [24]. Clinical studies and autopsies have demonstrated that people with heterozygous *ApoE ɛ4* are three times more likely to develop AD than noncarriers (odds ratio (OR) = 3.2), and people with a homozygous genotype of *ApoE ɛ4/4* are 14 times more likely to develop AD (OR = 14.9) [25]. *ApoE ɛ4* carriers have lower concentrations of A*β*1–42, higher total tau and phosphorylated-tau, and a higher degree of brain atrophy than individuals without *ApoE ɛ4* allele [26]. ApoE is thought to be involved in plaque formation, and this idea is supported by the finding that ApoE is involved in the deposition or clearance of A*β* by direct protein-to-protein interaction [27, 28]. We know that increased urinary AD7c-NTP levels are associated with tau-immunoreactive neurofibrillary tangles and amyloid-*β* (A*β*) deposition; similarly, the *ApoE* allele is also associated with hyperphosphorylated tau and A*β* deposition, which are the pathological hallmarks of AD [10, 27, 29, 30]. However, the relationship between the two biomarkers remains unclear. Assuming that these two biomarkers are highly consistent and can reflect the risk of disease development, it may be beneficial to monitor the progression of AD using urinary AD7c-NTP as a noninvasive biomarker. Therefore, this study investigated the correlation between urinary AD7c-NTP and *ApoE* genotype and explored whether urinary AD7c-NTP is affected by other factors that may lead to cognitive decline, further demonstrating the feasibility of urinary AD7c-NTP as a biomarker of AD.

## 2. Materials and Methods

### 2.1. Participants

This study is part of the Sino Longitudinal Study on Cognitive Decline (SILCODE) [31]. The study was approved by the Xuanwu Hospital Research Ethics Review Committee (ClinicalTrials.gov identifier: NCT03370744), and all participants signed their informed consent. A total of 329 cognitively normal right-handed Han Chinese subjects participated in the study, of which 151 were diagnosed with SCD and 178 were used as the cognitively normal controls (NC). All subjects were recruited through standardized public advertisements or memory clinics. SCD diagnosis was carried out by two experienced neurologists in the Department of Neurology, Xuanwu Hospital, Capital Medical University, based on the SCD-plus diagnostic framework of the Subjective Cognitive Decline Initiative [20, 23]. The inclusion criteria for SCD included Han Chinese nationality; right-handedness; older than 60 years; decline in memory as the primary symptom, rather than in any other cognitive domain; sustained cognitive decline in self-perception, independent of acute events, as compared with the previous healthy state; continuous concerns or worries associated with memory loss; cognitive function reported to be worse than that of others in the same age group; memory loss certified by an informed person; subject failed to meet the criteria for MCI or AD [32, 33].

The NCs were recruited from local communities through broadcast and online media advertising. The inclusion criteria for NC included Han Chinese nationality; right-handedness; older than 60 years; no memory or other cognitive decline complaints, with no concerns or worries about their cognition; normal scores in standardized neuropsychological tests, scale-adjusted for sex, age, and education; negative result for nervous system physical examinations; without any relevant medical histories or family histories; and accessory examinations revealed no diseases that could cause cognitive decline [31]. The exclusion criteria for all participants included congenital and acquired severe cognitive decline, MCI, AD, vascular cognitive dysfunction, or other dementia; a history of stroke, severe psychiatric disease, Parkinson's disease, multiple sclerosis, or brain tumor; alcohol or drug abuse; syphilis or acquired immune deficiency syndrome; severe liver and kidney dysfunction; severe hearing or visual impairment; and failure to cooperate with the study protocol.

### 2.2. Neuropsychological Assessment and Laboratory Measurements

All subjects underwent detailed medical history inquiries, neurological examinations, and medical system examinations and completed a neuropsychological assessment for diagnosis and differential diagnosis. Medical history collection included detailed inquiries about previous hypertension, diabetes, coronary heart disease, stroke, anemia, abnormal thyroid function, history of surgery under general anesthesia, history of COP, history of head trauma, and family history of dementia. Neuropsychological assessment scales included the Mini-Mental State Examination (MMSE), Montreal Cognitive Assessment-Basic (MoCA-B), Animal Verbal Fluency Test, Boston Naming Test (30 items), Auditory Verbal Learning Test-HuaShan (AVLT-H), Shape Trails (test A and test B), 17-item Hamilton Depression Rating Scale (HAMD), Hamilton Anxiety Scale (HAMA), and Functional Activities Questionnaire (FAQ). MMSE scores > 24 were considered normal for subjects with more than 6 years of education, while MMSE scores > 20 were considered normal for subjects with 1–6 years of education. When the subject was illiterate, an MMSE score > 17 was considered normal [34]. MoCA-B scores were considered normal when they were MoCA − B > 19 for the subjects with 0–6 years of education, >22 for those with 7–12 years of education, and>24 for those with 13 or more years of education [35].

All participants had blood samples collected in the morning after 8 hours with no food or water. Laboratory tests included a routine blood test, routine urine test, blood biochemistry, homocysteine, activated partial thromboplastin time, antibody tests for syphilis and human immunodeficiency virus, thyroid series, serum folic acid test, and serum vitamin B_12_ test, which were used to exclude other diseases that may lead to memory loss. *ApoE* gene testing was performed on all the subjects by Professor Yanning Cai. *ApoE* genotyping was performed by sequencing codons 112 and 158 of exon 4 of the *ApoE* gene [36].

### 2.3. Urinary AD7c-NTP Laboratory Detection

The levels of AD7c-NTP in urine samples were measured using the enzyme-linked immunosorbent assay AD7c-NTP kit (Anqun Biological Technology Co. Ltd., Shenzhen, China). All subjects collected clean midstream urine samples in the morning and placed them in Eppendorf tubes containing boric acid (2 g/L) as a preservative. Samples were centrifuged immediately and stored in a refrigerator at 4°C. The urine samples were observed visually. If the urine was cloudy or dark, it was discarded, and urine samples were collected the next morning. According to the manufacturer's instructions, the concentrated washing solution was diluted with distilled water at a ratio of 1 : 25. First, approximately 100 *μ*L of the sample was added, and the solution was incubated at 37°C for 60 min. The sample was then removed, the liquid on the plate was shaken off, and five consecutive wash steps were performed using phosphate-buffered saline (PBS) before the sample was patted dry. Next, 100 *μ*L of biotinylated rabbit anti-AD7c-NTP antibody was added and incubated at 37°C for 30 min. The reaction plate was removed, washed thoroughly with PBS, and patted dry. Next, 100 *μ*L of horseradish peroxidase-labeled avidin was added to the reaction plate, which was then sealed and incubated at 37°C for 30 min. After washing five times with PBS, 50 *μ*L of chromogenic reagents A and B were added in turn, mixed well, and incubated at 37°C for 15 min. Finally, the reaction was stopped by adding 50 *μ*L of sulfuric acid as the stop buffer and mixing well. A microplate reader was used to measure the absorbance (A value) at 450 nm wavelength, and the AD7c-NTP concentration was calculated according to the formula.

### 2.4. Classification of Demographic Characteristics and Blood ApoE Genotype

Education levels were grouped by high school level and divided into less than 12 years, 12 years, and more than 12 years of education. The nature of each subject's employment was classified into manual labor, mental labor, or mixed labor.

Previous clinical- or autopsy-based studies reported that AD risk is increased in people with genotypes *ApoE ɛ2/4*, *ApoE ɛ3/4*, and *ApoE ɛ4/4*; in contrast, AD risk is decreased in people with genotypes *ApoE ɛ2/2* and *ApoE ɛ2/3* [25]. Based on this information, all subjects were divided into four groups according to their genotype. Grade 1 included genotypes *ApoE ɛ2/2* and *ApoE ɛ2/3* (the risk of AD is reduced with these two genotypes, OR = 0.6 for each), grade 2 was made up of the genotype *ApoE ɛ3/3* (this genotype neither increases nor decreases the risk of AD), grade 3 included the *ApoE ɛ2/4* genotype (this genotype confers a mildly elevated risk of AD; OR = 2.6), and grade 4 was made up of the genotypes *ApoE ɛ3/4* and *ApoE ɛ4/4* (these genotypes confer a significantly increased risk of AD; OR = 3.2 and OR = 14.9; Table 1).

History of coronary heart disease (CHD) verified by medical documents and the diagnosis was based on the 2013 ESC guidelines for the diagnosis and management of stable coronary artery disease [37]. A diagnosis of diabetes was based on the American Diabetes Association criteria for elevated fasting blood glucose (≥7.0 mmol/L or ≥126 mg/dL) for patients with a history of diabetes [38]. Hypertension was defined as a clinical history of hypertension for more than 1 year, excluded secondary hypertension [39]. A history of stroke was defined as a history of cerebral infarction, cerebral hemorrhage, or subarachnoid hemorrhage and was certified by medical documents. Anemia was defined as hemoglobin below 120 g/L in men and 110 g/L in women. A history of hyperthyroidism and hypothyroidism, COP, general anesthesia, and head trauma, and a family history of dementia (FHD), were all self-reported and verified using medical or hospital records. Subjects with these histories were assigned to the positive group (+), while subjects without these histories were assigned to the negative group (–).

### 2.5. Statistical Analysis

All data were analyzed using the Statistical Package for Social Sciences (SPSS) v22.0 software. Data with continuous variables were expressed as the mean ± standard deviation (SD). Data for discontinuous variables were expressed as the median (interquartile range). Counting data were analyzed using the chi-square test. The two-independent-sample *t*-test was adopted to compare data between two groups, and the analysis of variance (ANOVA) was used to compare data among three or four groups. The Mann–Whitney *U* test was used to compare data for discontinuous variables, while the Kruskal–Wallis test for multiple comparisons was used for discontinuous variables. Further, Spearman correlation analysis was used to analyze the correlation between *ApoE* allele and urinary AD7c-NTP levels. A threshold of *p* < 0.05 was considered statistically significant.

## 3. Results

### 3.1. Participant Demographic Data

We recruited 329 right-handed Han Chinese subjects with an average age of 64.16 ± 6.43 years; 30.7% (*n* = 101) of the subjects were male (Table 1). Disease prevalence, including the prevalence of CHD, diabetes, hypertension, stroke, anemia, and thyroid dysfunction, is recorded in Table 1. Past medical history, such as COP, general anesthesia, head trauma, and family history of dementia, is also shown in Table 1. Personal history data, including years in education and nature of work, are shown in Table 1, and subjects were grouped according to their personal history or past medical history. *ApoE* genotype was detected and consisted of *ApoE ɛ2/2* (2 subjects), *ApoE ɛ2/3* (38 subjects), *ApoE ɛ3/3* (208 subjects), *ApoE ɛ2/4* (11 subjects), *ApoE ɛ3/4* (68 subjects), and *ApoE ɛ4/4* (2 subjects). The subjects were divided into four groups based on *ApoE* genotype (Table 1).

### 3.2. Urinary AD7c-NTP Levels by Years of Education and Nature of Work

According to the Kruskal–Wallis test, there were no significant differences in urinary AD7c-NTP levels among subjects with different years of education or nature of work (*p* > 0.05, Table 2).

### 3.3. Urinary AD7c-NTP Levels by Past Medical History and Family History of Dementia

In Table 2, there was a significant difference in urinary AD7c-NTP levels between subjects with a history of CHD and subjects without a history of this disease (*Z* = –2.854, *p* = 0.004). There was also a significant difference in urinary AD7c-NTP levels between subjects with and without a history of diabetes (*Z* = –2.725, *p* = 0.006). According to the Mann–Whitney *U* test, there were no significant differences in urinary AD7c-NTP levels in subjects with a family history of dementia or other medical histories such as hypertension, stroke, anemia, thyroid dysfunction, COP, general anesthesia, or head trauma (*p* > 0.05, Table 2).

### 3.4. Urinary AD7c-NTP Levels by SCD Diagnosis and ApoE Genotype

There was no significant difference in urinary AD7c-NTP levels between the NC [0.5164 (0.3667) ng/mL] and SCD [0.5483 (0.4838) ng/mL] groups (*p* > 0.05, Table 2). The Kruskal–Wallis test revealed that there were significant differences in urinary AD7c-NTP levels among subjects with different ApoE genotypes (*H* = 9.080, *p* < 0.05, Table 2). Furthermore, urinary AD7c-NTP levels in subjects with *ApoE ɛ3/4 and ApoE ɛ4/4* were significantly higher [0.6074 (0.6541) ng/mL] than in subjects without *ApoE ɛ4* [0.4368 (0.3392) ng/mL and 0.5287 (0.3656) ng/mL].

**Figure 1:**
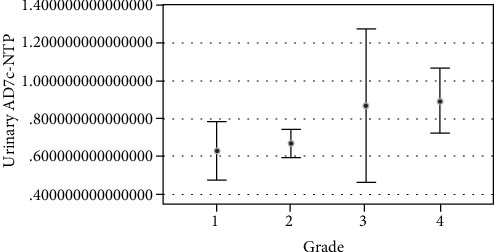
Correlation analysis between urinary Alzheimer-associated neuronal thread protein and *ApoE* allele grade. The line segment shows the 95% confidence interval.

Moreover, Spearman correlation analysis revealed that urinary AD7c-NTP levels were positively correlated with *ApoE* grade; that is, urinary AD7c-NTP levels increased with increased *ApoE* grade (*r* = 0.165, *p* = 0.003, Figure 1).

## 4. Discussion

The present study was designed to investigate the relationship between urinary AD7c-NTP levels and *ApoE ɛ4* alleles, as well as with other factors associated with cognitive decline in the cognitively normal population. Our study revealed that (i) urinary AD7c-NTP levels were not significantly different among subjects with different years of education and nature of work; (ii) there were significant differences in urinary AD7c-NTP between subjects with and without a history of CHD or diabetes; (iii) urinary AD7c-NTP levels were not significantly different in people with a family history of dementia or a history of hypertension, stroke, anemia, or thyroid dysfunction; and (iv) urinary AD7c-NTP levels in subjects with *ApoE ɛ4* were significantly higher than in subjects without *ApoE ɛ4*, and urinary AD7c-NTP levels increased with an increase in *ApoE* grade.

### 4.1. The Relationship between Urinary AD7c-NTP Levels, Personal History, and SCD Diagnosis

In the present study, urinary AD7c-NTP levels were not significantly different among subjects with different years of education or nature of work. These findings are in line with other reports that urinary AD7c-NTP is not affected by demographic factors or common chronic diseases [14]. Previous evidence suggests that being married and living in an urban environment can decrease the risk of cognitive impairment [40]. In the current study, although urinary AD7c-NTP levels were not increased in subjects with fewer years of education or who had worked in manual labor, the speed of cognitive decline affected by cognitive reserve remains a focus for neurologists.

In the present study, it was revealed that the levels of urinary AD7c-NTP were not higher in SCD patients compared with the NC group. This result is consistent with previous results from our research group [15]. The reason for these results may be that SCD is still a very early stage of the disease when neuropsychological test scores are normal; thus, tau protein may not be increased or detected at this stage [41]. Findings from the DELCODE study included a decline in memory and language in SCD, and SCD-plus features were associated with lower A*β*42 and a lower A*β*42/tau ratio but were not associated with total tau or p-tau-181 levels [42]. Furthermore, the DELCODE study reported lower CSF-A*β*42 levels and lower CSF-A*β*42/tau ratios in SCD patients than in healthy controls, while total tau and p-tau-181 levels were not elevated in the SCD group [42]. Urinary AD7c-NTP levels are a good indicator of tau protein levels; there is no increase in tau in SCD, and this evidence further supports our result of normal urinary AD7c-NTP levels in SCD patients. Urinary AD7c-NTP is a promising biomarker for AD, and its level is proportional to the degree of dementia [43]. SCD is still in the preclinical AD stage, without objective evidence of cognitive impairment, and this may be one of the reasons why urinary AD7c-NTP was not elevated in subjects with SCD.

### 4.2. The Relationship between Urinary AD7c-NTP Levels and Family History of Dementia or Past Medical History

Previous studies have reported that a family history of dementia increases the risk of developing dementia [44, 45]. In the current study, urinary AD7c-NTP levels were not different in people with or without a family history of dementia. This result suggests that a family history of dementia does not increase urinary AD7c-NTP levels. This may be because urinary AD7c-NTP is an indicator of tau, and there may not be any abnormal increase in tau in these subjects with a family history of dementia.

Another important finding was that urinary AD7c-NTP levels were elevated in people with a history of CHD and diabetes. CHD and diabetes are both risk factors for AD and dementia [40, 46]. Previous research has demonstrated that cholesterol metabolic disorder is a common cause and risk factor of CHD and AD [47]. There is evidence that *ApoE ɛ4* alleles and hyperlipidemia play a crucial role in the relationship between AD and CHD. Hyperlipidemia could aggravate coronary atherosclerosis and damage the blood–brain barrier, as well as promote A*β* protein production and tau deposition in the brain [47]. Diabetes increases the risk of AD by affecting glucose transmission to the brain and reducing glucose metabolism. It has been reported that glucose/lipid metabolism, oxidative stress, mitochondrial dysfunction, and protein changes in metabolic disorders caused by diabetes can all increase the prevalence of AD by promoting pathological changes in A*β* in diabetic patients [48]. Our results from the present study suggest that urinary AD7c-NTP is elevated in subjects with AD risk factors, and that urinary AD7c-NTP may therefore be a peripheral biomarker to predict AD risk factors.

Urinary AD7c-NTP levels were not elevated in people with a medical history of hypertension, stroke, anemia, or thyroid dysfunction in the current study. This result is also in accordance with earlier observations by our team, which revealed that urinary AD7c-NTP levels were not affected by common chronic diseases such as hypertension, stroke, dyslipidemia, renal insufficiency, cancer, chronic lung disease, chronic liver disease, or symptoms of depression [14]. The present study differs from the previous one in that we chose different diseases, but they are all chronic diseases that may cause cognitive decline. Hypertension and stroke are known risk factors for AD and dementia [40, 46]. A recent study reported that urinary AD7c-NTP is increased in hypertensive patients with cognitive decline; in contrast, the subjects in our study were from a population with a high risk of AD, but with normal cognition [12]. In our study, subjects with hypertension and normal cognition did not show elevated urinary AD7c-NTP. Anemia and high levels of hemoglobin are associated with an increased risk of AD [49]. Recent evidence suggests that hypothyroidism and hyperthyroidism are associated with AD, and hypothyroidism or hyperthyroidism may be one cause of cognitive impairment, including AD [50]. Although these diseases may increase the risk of cognitive decline, there is little evidence that these diseases are associated with increased tau protein. This may help to explain why there was no increase in urinary AD7c-NTP levels in subjects with a history of these diseases in the current study. COP, general anesthesia, and head trauma may also contribute to cognitive decline [51–53]. Cognitive impairment after COP is thought to be related to delayed encephalopathy caused by carbon monoxide, which is a symptom of frontal lobe dysfunction [51, 54]. A recent study reported that major surgery is associated with a small amount of long-term cognitive decline: cognitive decline after surgery was approximately double the level that it was before surgery [55]. Some studies have also demonstrated that about half of all patients with mild traumatic brain injury had long-term cognitive impairment and cognitive decline, including in learning and memory, attention, executive function, and processing speed [53]. Nevertheless, none of these causes of cognitive impairment involve neurodegeneration. Thus, in our study, we did not find elevated urinary AD7c-NTP levels in subjects with a history of COP, general anesthesia, or head trauma.

### 4.3. The Relationship between Urinary AD7c-NTP Levels and ApoE Genotype

The most important finding to emerge from the present analysis was that urinary AD7c-NTP levels were significantly higher in subjects with *ApoE ɛ4* genotypes than in subjects without *ApoE ɛ4* genotypes and that urinary AD7c-NTP levels increased with increased *ApoE* grade. This finding is consistent with that of previous studies [56], which have shown that urinary AD7c-NTP levels are elevated in MCI patients with *ApoE ɛ4* alleles [56].

Carrying the *ApoE ɛ4* allele is considered to be the primary genetic risk factor for sporadic AD [57]. Previous studies have confirmed that *ApoE ɛ4* prevalence is 51% in the cognitively normal population, 64% in patients with mild cognitive impairment, and 66% in patients with AD [58]. In particular, *ApoE ɛ4* carriers are more likely to develop AD at an earlier age than those without an *ApoE ɛ4* allele [59]. Previous studies have reported that heterozygous *ApoE ɛ4* carriers shift the risk curve to develop the disease 5 years earlier, while homozygous *ApoE ɛ4/4* carriers shift it to develop the disease 10 years earlier, and *ApoE ɛ2* carriers shift it to develop the disease 5 years later [59, 60]. Furthermore, evidence has revealed that *ApoE* influences A*β* deposition in a dose- and isoform-specific fashion (*ɛ*4 > *ɛ*3 > *ɛ*2) [61]. Therefore, in this study, we ranked the *ApoE* alleles according to their AD risk.

Urinary AD7c-NTP levels were higher in subjects with *ApoE ɛ4* than in subjects without *ApoE ɛ4* in the present study. This result may be because both *ApoE* and urinary AD7c-NTP are related to tau and A*β* deposition. Substantial evidence suggests that the *ApoE ɛ4* allele is related to increased A*β* deposition, rapid thinning of the cortex, and accelerated cognitive decline, while the *ApoE ɛ2* allele is related to a decrease in A*β* deposits, slower thinning of the cortex, and slower cognitive decline [62–65]. Reports from experiments on animals show that the *ApoE ɛ4* allele disrupts memory function in rodents, and further studies have indicated that fragments of the *ApoE ɛ4* allele may contribute to both plaque and tangle formation [66]. It has been demonstrated that the *ApoE* genotype can affect tau neuropathological changes in AD patients [47]. In the current study, there was a positive correlation between urinary AD7c-NTP levels and *ApoE* grades. There is evidence that *ApoE ε*4 is associated with a higher density of paired helical filament tau tangles, while *ApoE ε*2 is associated with fewer paired helical filament tau tangles in AD patients with A*β* [61]. In addition, experiments on animals have shown that the *ApoE* allele affects tau pathogenesis and tau-mediated neurodegeneration [67].

*ApoE* allele and urinary AD7c-NTP levels are both promising biomarkers of AD. Previous studies have shown that the combined detection of *ApoE ε*4 and urinary AD7c-NTP is a reliable biomarker for the early diagnosis of AD and that the predictive value is significantly increased compared with the detection of either one individually [56, 68]. The high consistency of these two biomarkers also provides a new way for us to understand and think about AD. The combination of two biomarkers may help the early diagnosis of AD and improve the diagnostic accuracy of AD and be better than one biomarker alone.

There are, however, some limitations to this study. First, subjects with normal cognition were recruited, and the correlation between urinary AD7c-NTP levels and *ApoE* allele in patients with abnormal cognition, such as with MCI or AD, should be further analyzed. Second, because of the low incidence of *ApoE ɛ2/4* in the population, this study included only 11 subjects with *ApoE ɛ2/4* out of 329 cognitively normal subjects; this genotype was relatively rare compared with other genotypes. Third, a longitudinal, multicenter, large study is needed to observe dynamic changes in AD7c-NTP levels in urine.

## 5. Conclusions

There were significant differences in urinary AD7c-NTP levels between subjects with and without a history of CHD or diabetes. Urinary AD7c-NTP levels were positively correlated with *ApoE* grade in the cognitively normal population. The relationship between AD risk factors and urinary AD7c-NTP levels may provide a new way for us to understand AD and urinary AD7c-NTP.

## Figures and Tables

**Table 1:** Demographics and clinical features of the study participants.

Variables	Subject group	Age (y)	Male: female	No. cases	Percent (%)
Education level	<12	64.62 ± 5.24	23 : 82	105	31.92
	=12	64.28 ± 6.22	24 : 50	74	22.49
	>12	63.78 ± 7.25	54 : 96	150	45.59
*ApoE* allele	Grade 1 (2/2 + 2/3)	64.23 ± 4.99	13 : 27	40	12.16
	Grade 2 (3/3)	64.21 ± 6.53	67 : 141	208	63.22
	Grade 3 (2/4)	64.55 ± 5.82	4 : 7	11	3.34
	Grade 4 (3/4 + 4/4)	63.91 ± 7.04	17 : 53	70	21.28
Nature of work	Mental labor	64.67 ± 6.51	70 : 150	220	66.87
	Manual labor	62.54 ± 5.43	10 : 27	37	11.25
	Mixed labor	63.43 ± 6.52	21 : 51	72	21.88
CHD	+	66.23 ± 5.91	11 : 11	22	6.69
	—	64.01 ± 6.45	90 : 217	307	93.31
Diabetes	+	65.38 ± 6.06	16 : 21	37	11.25
	—	64.01 ± 6.47	85 : 207	292	88.75
Hypertension	+	65.04 ± 6.18	46 : 64	110	33.43
	—	63.72 ± 6.52	55 : 164	219	66.57
Stroke	+	65.04 ± 7.11	8 : 16	24	7.29
	—	64.09 ± 6.38	93 : 212	305	92.71
Anemia	+	62.69 ± 7.56	1 : 15	16	4.86
	—	64.24 ± 6.37	100 : 213	313	95.14
Thyroid dysfunction	+	63.29 ± 8.22	7 : 14	21	6.38
	—	64.22 ± 6.30	94 : 214	308	93.62
COP	+	65.94 ± 6.25	16 : 34	50	15.20
	—	63.84 ± 6.42	85 : 194	279	84.80
General anesthesia	+	64.51 ± 6.03	20 : 56	76	23.10
	—	64.06 ± 6.56	81 : 172	253	76.90
Head trauma	+	67.27 ± 4.80	4 : 7	11	3.34
	—	64.05 ± 6.46	97 : 221	318	96.66
FHD	+	63.24 ± 5.87	17 : 65	82	24.92
	—	64.47 ± 6.59	84 : 163	247	75.08
Diagnosis	SCD	63.77 ± 6.25	43 : 108	151	45.90
	NC	64.49 ± 6.58	58 : 120	178	54.10

Key: CHD: history of coronary heart disease; COP: carbon monoxide poisoning; FHD: family history of dementia; SCD: subjective cognitive decline; NC: normal controls.

**Table 2:** Comparison of urinary AD7c-NTP levels in different demographic characteristics groups, nonneurological disease groups, and different *ApoE* allele grade groups.

Variables	Subject group	AD7c-NTP (ng/mL)	*H/Z*	*p*
Education level	<12	0.5562 (0.5261)	0.233	0.890
	=12	0.5165 (0.3971)		
	>12	0.5368 (0.3310)		
Nature of work	Mental labor	0.5184 (0.3681)	2.902	0.234
	Manual labor	0.6311 (0.6305)		
	Mixed labor	0.5711 (0.3899)		
CHD	+	0.7921 (0.7347)	-2.854	0.004^∗^
	—	0.5246 (0.3823)		
Diabetes	+	0.6355 (0.5824)	-2.725	0.006^∗^
	—	0.5211 (0.3889)		
Hypertension	+	0.5232 (0.4389)	-0.014	0.989
	—	0.5381 (0.3925)		
Stroke	+	0.6267 (0.6099)	-1.738	0.082
	—	0.5315 (0.3880)		
Anemia	+	0.5784 (0.4786)	-0.079	0.937
	—	0.5322 (0.4091)		
Thyroid dysfunction	+	0.6241 (0.8910)	-0.245	0.806
	—	0.5322 (0.3897)		
COP	+	0.5964 (0.5298)	-0.392	0.695
	—	0.5265 (0.3918)		
General anesthesia	+	0.5665 (0.4077)	-0.776	0.438
	—	0.5265 (0.4035)		
Head trauma	+	0.4969 (0.2551)	-0.274	0.784
	—	0.5342 (0.4212)		
FHD	+	0.5417 (0.3776)	-0.442	0.658
	—	0.5322 (0.4428)		
Diagnosis	SCD	0.5483 (0.4838)	-1.359	0.174
	NC	0.5164 (0.3667)		
*ApoE* allele	Grade 1 (2/2 + 2/3)	0.4368 (0.3392)		
	Grade 2 (3/3)	0.5287 (0.3656)	9.080	0.028^∗^
	Grade 3 (2/4)	0.5580 (0.9201)		
	Grade 4 (3/4 + 4/4)	0.6074 (0.6541)		

Key: AD7c-NTP: Alzheimer-associated neuronal thread protein; CHD: history of coronary heart disease; COP: carbon monoxide poisoning; FHD: family history of dementia; SCD: subjective cognitive decline; NC: normal controls; Data in AD7c-NTP were shown in median (interquartile range).

## Data Availability

The data used to support the findings of the present research are available from the corresponding authors once requested.
